# Supplementary material for: Direct Cardiac Epigenetic Reprogramming through Codelivery of 5′Azacytidine and miR-133a Nanoformulation
Source: Int J Mol Sci. 2022 Dec 2;23(23):15179. doi: 10.3390/ijms232315179 (PMC9739153; doi:10.3390/ijms232315179)
Supplement: Supplementary file 1 [file ijms-23-15179-s001.zip › ijms-1829268-SI.pdf]

Supplementary material

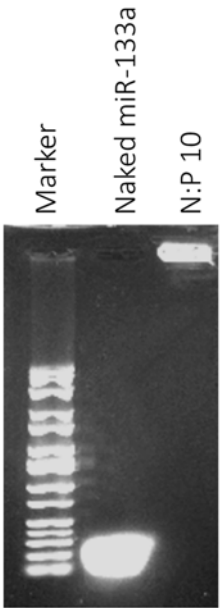

Figure S1. : Gel retardation assay of N:P ratio 10. The polyplexes were formed by self-assembly mechanism. Briefly, miR-133a was added to PEI (1mg/mL) at a N:P ratio of 10 and incubated for 30 mins at room temperature. Following the incubation, the polyplexes were loaded on 1% agarose gel with gel red and electrophoresed for 100 V for 30 mins. The bands were visualized using ultraviolet illuminator. The formation of polyplexes were confirmed by the retardation of miR-133a bands.

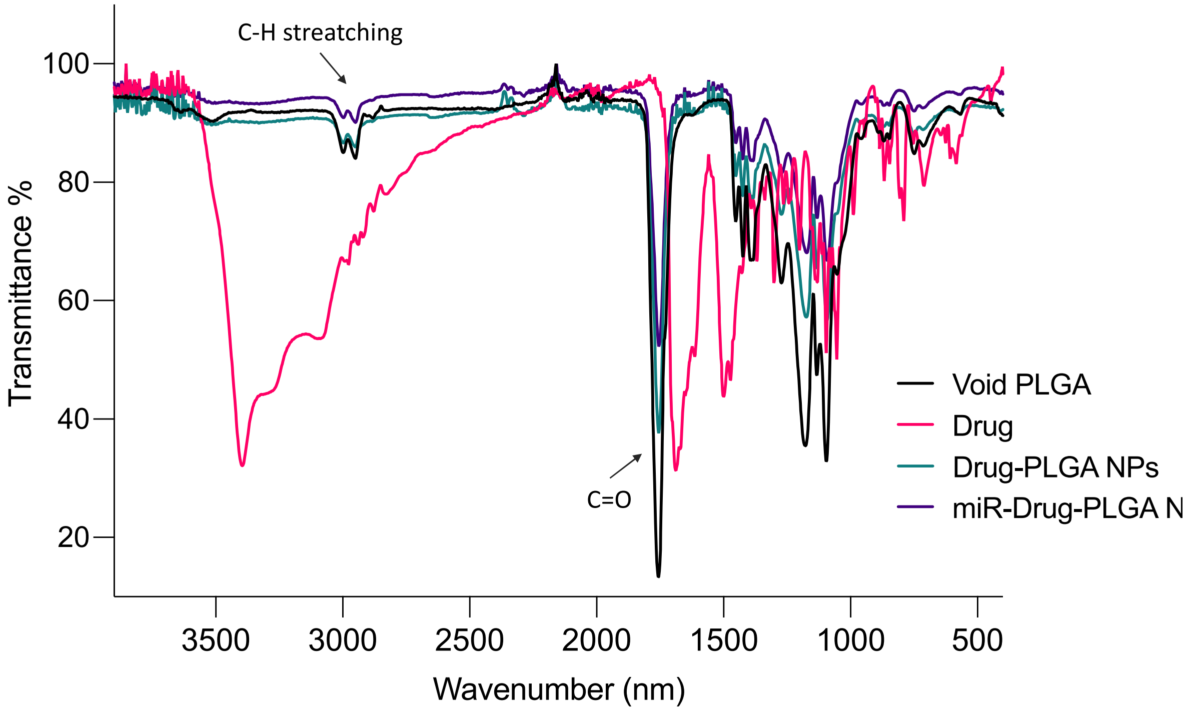

Figure S2. FTIR spectra for Void PLGA NPs, Drug, PLGA-AZA NPs and PLGA-PEI-miR-133a-AZA NPs.

Table S1. Main peaks of PLGA NPs FTIR spectra.

Peak position (Wavenumber cm^{-1})	Assignments
3000	Asymmetric CH_3 stretching
2952	Asymmetric CH_2 stretching
1754	Carbonyl C=O stretching (alkyl groups)
1454	C-H bending of CH_3
1425	CH bending of O-CH, (of glycolic acid) and OH in plane bending
1392	C-H bending of CH_3
1268	C-O stretching of carboxylic acid
1180	C-C-O stretching from ester
1093	O-C-C stretching from ester
958	OH bending

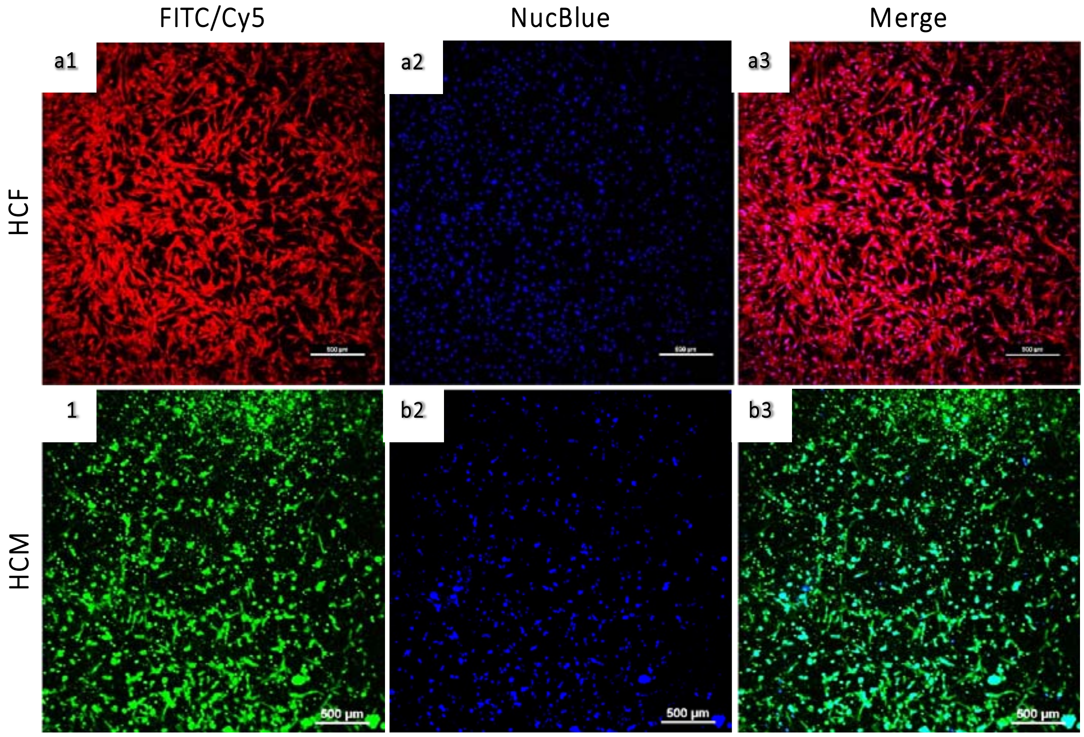

Figure S3. Cellular Uptake of PEI-Cy5miR-133a (a1-a3) and PLGA-FITC (b1-b3) and Nanospheres using confocal microscopy in HCF and HCM respectively. The nanospheres were labelled with FITC (green/Cy5(Red)). The cells were incubated with PLGA-FITC NPs/Cy5-miR-133a for 4 h to evaluate cellular internalization of the polymer into the cell. (a1) shows PEI-Cy5-miR-133a uptake by HCF cells. (b1) shows FITC particle internalization by HCM cells. (Scale bar= 500 µm).

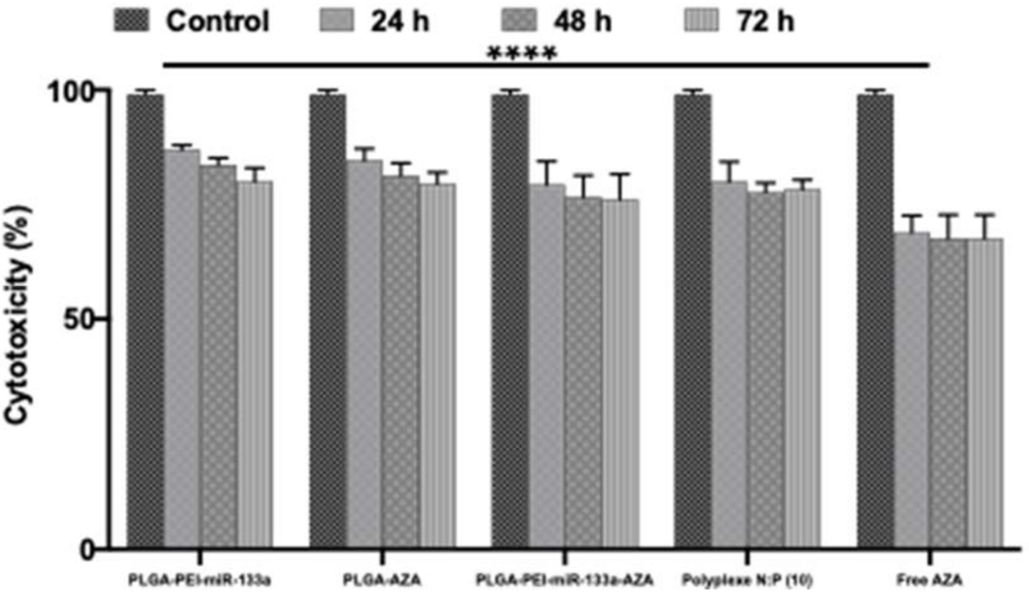

Figure S4. *Cell viability were evaluated to assess the compatibility of HCM treated with PLGA NPs* PLGA-PEI-miR-133a, PLGA-AZA and PLGA-PEI-miR-133a-AZA at a concentration of 100 µ g/mL in comparison with miR-133a polyplexes (50nM was used to for complex with PEI at an N:P ratio 10) , free drug (1 mg/mL) and control for 24 h, 48 h and 72 h. The cell viability of all PLGA NPs were 80% viable at all time point. The ratios of PLGA NPs at all doses and time points demonstrated that the experiment was extremely statistically significant, with p < 0.05 (* p ≤ 0.05 ** p ≤ 0.01 *** p ≤ 0.001 **** p ≤ 0.0001). experiment was extremely statistically significant, with $p < 0.05$ (* $p \leq 0.05$ ** $p \leq 0.01$ *** $p \leq 0.001$ **** $p \leq 0.0001$).
